# Multifaceted interplay between lipophilicity, protein interaction and luminescence parameters of non-intercalative ruthenium(II) polypyridyl complexes controlling cellular imaging and cytotoxic properties

**DOI:** 10.1007/s00775-014-1187-5

**Published:** 2014-08-26

**Authors:** Olga Mazuryk, Katarzyna Magiera, Barbara Rys, Franck Suzenet, Claudine Kieda, Małgorzata Brindell

**Affiliations:** 1 Department of Inorganic Chemistry, Faculty of Chemistry, Jagiellonian University, Ingardena 3, 30-060 Krakow, Poland; 2 Department of Organic Chemistry, Faculty of Chemistry, Jagiellonian University, Ingardena 3, 30-060 Krakow, Poland; 3 Institute of Organic and Analytical Chemistry, University of Orléans, UMR-CNRS 7311, rue de Chartres, BP 6759, 45067 Orléans cedex 2, France; 4 Centre de Biophysique moléculaire, CNRS, rue Charles Sadron, 45071 Orléans cedex 2, France

**Keywords:** Ruthenium polypyridyl complexes, Cytotoxicity, Optical imaging, Luminescence, Protein binding

## Abstract

Here, we examine the photophysical properties of five ruthenium(II) complexes comprising two 4,7-diphenyl-1,10-phenanthroline (dip) ligands and functionalized bipyridine (R_1_bpy-R_2_, where R_1_ = H or CH_3_, R_2_ = H, CH_3_, COO^−^,4-[3-(2-nitro-1*H*-imidazol-1-yl)propyl] or 1,3-dicyclohexyl-1-carbonyl-urea) towards development of luminescence probes for cellular imaging. These complexes have been shown to interact with albumin and the formed adducts exhibited up to eightfold increase in the luminescence quantum yield as well as the average lifetime of emission. It was demonstrated that they cannot bind to DNA through the intercalation mode and its luminescence in the presence of DNA is quenching. Cell viability experiments indicated that all complexes possess significant dose-dependent cytotoxicity (with IC_50_ 5–19 μM) on 4T1 breast cancer cell line and their anti-proliferative activity correlates very well with their lipophilicity. Cellular uptake was studied by measuring the ruthenium content in cells using ICP-MS technique. As expected, the better uptake is directly related to higher lipophilicity of doubly charged ruthenium complexes while uptake of monocationic one is much lower in spite of the highest lipophilicity. Additionally staining properties were assessed using flow cytometry and fluorescence microscopy. These experiments showed that complex with 1,3-dicyclohexyl-1-carbonyl-urea substituent exhibits the best staining properties in spite of the lowest luminescence quantum yield in buffered solution (pH 7.4). Our results point out that both the imaging and cytotoxic properties of the studied ruthenium complexes are strongly influence by the level of internalization and protein interaction.

**Electronic supplementary material:**

The online version of this article (doi:10.1007/s00775-014-1187-5) contains supplementary material, which is available to authorized users.

## Introduction

Ruthenium polypyridyl complexes have been studied as structure- and site-specific DNA probes and nucleus imaging agents in biological systems, since the interaction of [Ru(bpy)_2_(dppz)]^2+^ (bpy: 2,2′-bipyridine, dppz: dipyrido[3,2-a:2′3′-c]phenazine) complexes with DNA through intercalation revealed significant enhancement of the luminescent intensity, the so-called “light switch” effect [1, 2]. Despite high DNA-binding constant (>10^6^ M^−1^) [3, 4], ruthenium complexes of the type [RuL_2_(dppz)]^2+^(L = bpy, phen: phenanthroline, dip: 4,7-diphenyl-1,10-phenanthroline) demonstrate cellular internalization (staining of cytoplasm) with limited nuclear accumulation in live cells [5]. One of the possible reasons for this is impermeability of nucleus membrane of live cells for ruthenium complexes. For a probe to be selective towards nucleus, among others it should have a cationic but also amphipathic character with the logarithm of water–octanol partition coefficient (log *P*_o/w_) in the range −5 to 0 to facilitate crossing both cellular and nucleus membranes, high base strength (p*K*_*a*_ > 10) to exclude localization in lysosome and finally a planar aromatic system for intercalation [6]. Some researchers have tried to optimize the polypyridyl ligands to reach selective accumulation in cellular DNA, e.g., by modification of dppz ligand with a nuclear targeting peptide chain [7–9] or with substituents increasing its hydrophobicity [2]. Recently the development of the ruthenium complexes toward their application in optical imaging of cells in hypoxia [10, 11] or as cytotoxic agents selectively activated in hypoxic cells [12] shows a new direction in design and great potency of this type of compounds. One of the most interesting research aspects is a dual imaging and therapeutic application of ruthenium polypyridyl complexes [10, 11, 13]. In this context an appropriate modification of polypyridyl ligands through the introduction of different substituents can tune cytotoxic and luminescent properties of ruthenium complexes.

**Scheme 1:**
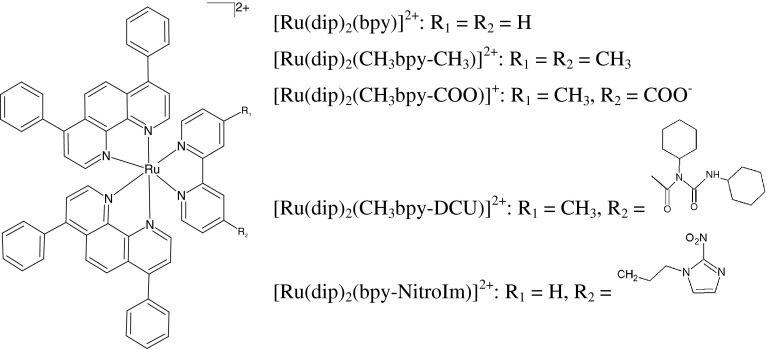
The studied ruthenium complexes

The principal purpose of the present study is to show the multifaceted relationship between lipophilicity, protein interaction and luminescence properties of ruthenium(II) complexes affecting cell imaging and cytotoxic properties. The system chosen for this study as illustrated in Scheme 1, is the family of the ruthenium(II) complexes comprising two dip ligands and one bpy ligand, which, in turn, possesses various substituents at 4 and 4′ positions. The [Ru(dip)_2_(bpy)]^2+^, [Ru(dip)_2_(CH_3_bpy-CH_3_)]^2+^ and [Ru(dip)_2_(CH_3_bpy-COO)]^+^(at pH > 5 the carboxylic group is deprotonated) are well known from the literature [14], we have recently published the synthesis of [Ru(dip)_2_(bpy-NitroIm)]^2+^ [11] while the formation of [Ru(dip)_2_(CH_3_bpy-DCU)]^2+^ is described in this work. These complexes have gained our attention since they are not expected to have intercalative properties as confirmed by previous work [11, 15] and these studies, therefore the cellular DNA is not postulated as their target. Moreover, the “light switch” effect in the presence of the DNA is not observed. This is in contrast to numerous studies for ruthenium polypyridyl complexes [3, 16–18]. The selected substituents tune lipophilic and photophysical properties. To demonstrate the interplay between physicochemical/photophysical properties and biological activity, we analyze the cytotoxicity and uptake of the studied compounds using 4T1 breast cancer cell line as well as the luminescence emitted by cells arising from ruthenium complex accumulation. We also show that the luminescence properties of these ruthenium complexes strongly depend on the interaction with albumin, which suggests that in cells the interaction with proteins can alter their imaging properties as well.

## Materials and methods

### Synthetic procedures

4,7-Diphenyl-1,10-phenanthroline (dip), 2,2′-bipyridine(bpy) and 4,4′-dimethyl-2,2′-bipyridine(CH_3_bpy-CH_3_) were purchased from Sigma-Aldrich. 4-carboxy-4′-methyl-2,2′-bipyridine(CH_3_bpy-COOH) and 4-[3-(2-nitro-1*H*-imidazol-1-yl)propyl]-2,2′-bipyridine (bpy-NitroIm) were prepared according to the published procedures [11, 19]. Ruthenium complexes of the type [Ru(dip)_2_L]Cl_2_ where L denotes bpy, CH_3_bpy-CH_3_, CH_3_bpy-COOH or bpy-NitroIm were prepared following the literature procedures [11, 19]. The purity of the synthesized complexes was checked by HPLC and MS analyses.

[Ru(dip)_2_(1,3-dicyclohexyl-1-[4-carbonyl-(4′-methyl-2,2′-bipyridyl)]-urea)]Cl_2 _([Ru(dip)_2_(CH_3_bpy-DCU)]Cl_2_) was synthesized as follows. To a stirred solution of [Ru(dip)_2_CH_3_bpy-COOH]Cl_2_ (40 mg, 0.038 mmol) in dry CH_2_Cl_2_ (3 ml) with triethylamine (0.15 ml, 1 mmol), solution of *N*,*N’*-dicyclohexylcarbodiimide (16 mg, 0.077 mmol) in dry CH_2_Cl_2_ (2 ml) was added. The mixture was stirred at room temperature for 48 h and then the solvent was removed under reduced pressure. The resulting solid was chromatographed on aluminium oxide using chloroform/methanol (50:1) solution as the eluent to afford final compound (12 mg, 27 %) as an orange solid. ^1^H NMR (600 MHz, CDCl_3_): *δ* 0.95–1.17 (m, 4H), 1.32–1.70 (m, 12H), 1.80 (m, 2H), 2.14 (bd, J 12.0, 2H), 2.63 (s, 3H), 3.33 (m, 1H), 4.31 (tt, J 3.4, 12.1, 1H), 7.42 (dd, J 1.0, 5.8, 1H), 7.49–7.62 (m, 1H), 7.65 (d, J 5.5, 1H), 7.71 (m, 3H), 7.75 (dd, J 1.6, 5.8, 1H), 7.78 (d, J 5.5, 1H), 7.88 (d, J 5.4, 1H), 7.95 (d, J 5.5, 1H), 8.07 (d, J 5.9, 1H), 8.09 (d, J 5.5, 1H), 8.16–8.25 (m, 4H), 8.30 (d, J 5.4, 1H), 8.48 (d, J 5.5, 1H), 8.77 (d, J 5.6, 1H), 9.00 (s, 1H), 9.30 (s, 1H), 9.49 (d, J 8.1, 1H) ppm. ^13^C NMR (150 MHz, CDCl_3_): *δ* 21.42, 25.05, 25.16, 25.46, 26.01, 30.71, 31.04, 31.45, 31.64, 50.96, 55.35, 123.11, 125.75, 125.84, 126.21, 126.38, 126.40, 126.64, 126.91, 127.45, 127.84, 128.75, 128.88, 128.90, 129.06, 129.12, 129.23, 129.28, 129.39, 129.64, 129.70, 129.74, 129.78, 129.97, 130.03, 135.23, 135.31, 135.37, 135.44, 146.12, 147.62, 147.98, 148.54, 148.58, 149.01, 149.30, 149.34, 149.55, 150.91, 151.09, 151.15, 151.41, 151.92, 152.73, 153.71, 154.18, 155.97, 157.41, 164.70 ppm. HRMS: calcd. for C_73_H_64_N_8_O_2_Ru [M^2+^] 593.2058, found 593.2092 (Fig. S1A). IR-ATR: 3354w, 3194w, 3054w, 2928m, 2853w, 2157w, 1971w, 1689m, 1649s, 1621s, 1533m. HPLC: *t*_R_ = 6.96 min, HILIC, acetonitrile/ammonium acetate (0.1 M), gradient from 95:5 to 1:1 (Fig. S1B).

### Spectroscopic measurements

UV–Vis absorption spectra were recorded on a Perkin Elmer Lambda 35 spectrophotometer. Luminescence measurements were performed on a spectrofluorimeter Perkin Elmer LS55. The spectra were recorded at the room temperature in aqueous solution containing small amount of DMSO (<0.008 % v/v). The emission spectra were recorded between 470 and 860 nm upon excitation at 463 nm. The average of three scans was subjected to smoothing. For determination of the quantum yield of luminescence (*Φ*), aqueous solutions of [Ru(bpy)_3_]^2+^ with a small amount of DMSO (<0.008 % v/v) were used as standards (*Φ* = 0.028 [14] and 0.042 [20] for air-equilibrium and deoxygenated conditions, respectively). The spectra were recorded for ruthenium complexes at the concentration less than 0.05 absorbance unit at the excitation wavelength. Values were calculated according to the following equation [21]:

$$\varPhi = \varPhi_{\text{ref}} \times \, \left[ {A_{\text{ref}} /A} \right] \, \times \, \left[ {I/I_{\text{ref}} } \right] \, \times \, \left[ {n^{2} /n_{\text{ref}}^{2} } \right],$$

where *I* is the integrated intensity of luminescence, *A* is the optical density, and *n* is the refractive index, ref refers to the values for reference. The mean value from minimum three independent experiments was calculated.

The luminescence lifetime measurements were performed with a single photon counting technique using Fluorolog-3, Horiba Jobin Yvon. The excitation wavelength was set at 464 nm (NanoLed Diodes) and the average lifetime of luminescence was monitored at 621 nm. Luminescence decays were collected with 1,000 counts in the peak. The instrument response functions were measured using a light scattering solution of Ludox (colloidal silica, Sigma-Aldrich). Experiments were conducted at room temperature. The DAS6 software (HORIBA Scientific) was used for deconvolution of the obtained decays and for calculation of the lifetime values. The quality of the fit was judged by the *χ* ^2^ parameter (the goodness of fit evaluation). One-exponential fit was determined to be an optimal description of the obtained results for the ruthenium compounds.

### Protein-binding experiments

The protein stock solution was prepared by dissolving human serum albumin (HSA) in water and its concentration was determined spectrophotometrically from the molar absorptivity of 4.4 × 10^4^ cm^−1^ M^−1^ at 280 nm [22–25]. The emission spectra were recorded between 305 and 500 nm upon excitation at 295 nm resulting in selective excitation of tryptophan residue of HSA. The average of three scans was subjected to smoothing and the fluorescence intensities were corrected due to dilution effects. Protein-binding experiments were conducted by measuring fluorescence spectra of protein solution (1 μM) in the presence of different amounts of ruthenium compounds (0–10 μM) in PBS buffer pH 7.4 at 37 °C. Ru–protein solutions were allowed to incubate for 5 min before the emission spectra were recorded. The quantum yield and lifetime of luminescence for ruthenium complexes in the present of HSA (1 μM) was measured using the same procedure as described for ruthenium complexes alone (the HSA/Ru-complex ratio is given in the figure caption).

### DNA-binding experiments

Calf thymus deoxyribonucleic acid was purchased from Sigma-Aldrich and its stock solution was prepared by dissolving of solid DNA in water. DNA concentrations per nucleotide were determined by absorption spectroscopy using the molar absorption coefficient of 6,600 M^−1^cm^−1^ at the wavelength of 260 nm [4]. DNA-binding experiments were performed in 0.05 M Tris/HCl buffer (pH 7.4) at 37 °C. The absorption titration experiments were performed by using fixed concentration of ruthenium compound (10 µM) until the absorption spectra did not change with increasing DNA concentration. Ruthenium–DNA solutions were allowed to incubate for 5 min before the spectra were recorded. The intrinsic DNA-binding constant was calculated from the following Eq. [4].

$$\frac{{\left[ {\text{DNA}} \right]}}{{\varepsilon_{\text{a}} - \varepsilon_{\text{f}} }} = \frac{{\left[ {\text{DNA}} \right]}}{{\varepsilon_{\text{a}} - \varepsilon_{\text{f}} }} + \frac{1}{{K_{\text{b}} \left( {\varepsilon_{\text{b}} - \varepsilon_{\text{f}} } \right)}},$$

where [DNA] is the total DNA concentration in nucleotides, *ε*_a_, *ε*_b_, *ε*_f_ are the apparent absorption coefficients of A/[ruthenium complex] of the MLCT absorption band at a given DNA concentration, fully bound and free ruthenium complex, respectively, *K*_b_ is binding constant.

The emission titration studies were performed by using fixed concentration of ruthenium compound (3 µM). The DNA aliquots were added and after 5 min of incubation luminescence spectra upon excitation at 463 nm were measured. The average of three scans was subjected to smoothing and the luminescence intensities were corrected due to dilution effects.

### Determination of lipophilicity

The lipophilicity of the ruthenium(II) complexes, which is referred to log *P*_o/w_ (*n*-octan-1-ol/water partition coefficient), was measured as following. Ruthenium complexes were dissolved in *n*-octan-1-ol (to mM concentration), then solutions were added to water and the mixtures were stirred sufficiently for partitioning at 25 °C for 24 h. After that the mixtures were left for equilibration for another 2 h. The concentration of the compounds in the water phase was measured spectrophotometrically and *P*_o/w_ value was calculated according to the equation: $P_{\text{O/w}} = \frac{{c_{\text{octanol}}^{\text{before}} - c_{\text{water}} }}{{c_{\text{water}} }}$, where $c_{\text{octanol}}^{\text{before}}$ is an initial concentration of ruthenium complex, *c*_water_ denotes final concentration in water [5]. The experiment was conducted in triplicates.

### Cell culture, cytotoxicity and apoptosis assays

4T1 breast cancer cell line was cultured in RPMI-1640 (Gibco Invitrogen) with 10 % fetal bovine serum (FBS, the bovine serum albumin is a major component), 1 % penicillin and streptomycin and 0.2 % fungizone. Cells were routinely cultured at 37 °C in a humidified incubator in 5 % CO_2_ atmosphere. Cell viability was measured using Alamar Blue assay. Cells were seeded on 96-well plate with density of 10^4^ cells per cm^2^ and cultured for 24 h in medium with or without 2 % serum. Then cells were incubated with various concentrations of ruthenium compounds for 24 h in the dark. All used ruthenium complexes were freshly diluted in DMSO and then added to the appropriate medium to obtain the applied concentrations. The final DMSO concentration was kept constant at 0.05 % (v/v). Next cells were washed with PBS and incubated in AlamarBlue solution (21 times diluted in PBS) for 3 h. Alamar Blue test is based on the reduction of blue and non-fluorescent subtract (resazurin) to a pink and highly fluorescent product (resorufin) by the alive cells. Extract mechanism of the reduction is still unknown, but it is postulated that reduction occurs by mitochondrial or cytoplasmic enzymes, such as NADH dehydrogenase or diaphorase. It is still not known whether this process occurs intracellularly, at the plasma membrane surface or just in the medium as a chemical reaction [26, 27]. The cell viability was quantified at 605 nm using 560 nm excitation light (VICTOR 3V multilabel plate readers, PerkinElmer). Experiments were performed in triplicates and each experiment was performed at least three times to get the mean values ± standard deviation. The viability was calculated with regard to the untreated cells control. The IC_50_ values were determined using Hill equation (Origin 9.0) [28]:

$$y = y_{0} + \frac{{\left( {y_{100} - y_{0} } \right)\left[ c \right]^{H} }}{{\left[ {{\text{IC}}_{50} } \right]^{H} + \left[ c \right]^{H} }}$$

The apoptosis was investigated using Hoechst 33258 staining method [29, 30]. 4T1 cells were seeded into 96-well plate with a density of 2 × 10^4^ cells per cm^2^ and cultured in the full medium for 24 h. The medium was removed and replaced with medium containing various concentrations of the ruthenium complexes. Cells were incubated with compounds for 24 h, then washed with ice-cold PBS, fixed with formalin (4 %). Cell nuclei were counterstained with Hoechst 33258 (10 µg/ml in PBS) for 15 min. Cells were then observed and imaged by an AxioVert 200M fluorescence microscope (Carl Zeiss).

### Ruthenium uptake measured by ICP-MS

Cells were seeded on a 6-well plate with a density of 3 × 10^5^ cells per cm^2^. 24 h after the incubation ruthenium complexes were added at 2 µM concentration. Cells were incubated in medium without serum for 24 h. After incubation cells were washed twice with PBS, detached by trypsin (trypsin/EDTA from Gibco) treatment, diluted in PBS and counted. Cells were digested in concentrated nitric acid overnight at room temperature and then diluted with water. The ruthenium content of the sample was determined by the inductively coupled plasma mass spectrometry (ICP-MS). Results were calculated as ruthenium concentration per cell (assuming the average volume of cell was 1.7 pL [31]).

### Ruthenium uptake measured by flow cytometry

4T1 cells were seeded in a 24-well plate with a density of 2 × 10^5^ cells per cm^2^. 24 h after the seeding Ru(II) compounds were added at 2 µM concentration and incubated for 24 h in medium with or without serum (2 %). Then cells were washed with PBS, detached by trypsin treatment and analyzed by BDLSR cytometer with an excitation wavelength of 488 nm and an emission wavelength of 575 ± 13 nm. The luminescence intensity of the control cells in the tested conditions was found to be negligible.

### Imaging

4T1 cells were seeded on the black 96-well plate with a transparent bottom with a density of 10^4^ cells per cm^2^ 24 h prior the staining. Next cells were incubated with 1 µM ruthenium complexes in medium with serum (2 %) for 24 h. After incubation cells were washed with PBS and images were acquired using an AxioVert 200M fluorescence microscope (Carl Zeiss). Illumination system Colibri with 4 LED excitation diodes (365, 470, 530 and 625 nm) was applied as source of fluorescence and cube filter with excitation wavelengths 470 and 555 nm, beam splitter 560 nm, emission range 575–640 nm was used.

## Results and discussion

### Photophysical characterization of the ruthenium(II) complexes

The absorption spectra of all studied ruthenium compounds (Fig. S2) possess an arrow and intense band at 278 nm assigned to a spin allowed ^1^LC (^1^ *π* → *π* ^*^bpy-R-centered) transition, the shoulder at 314 nm originating from a ^1^LC transition of phenanthroline moiety and two not well separated bands at 400–500 nm attributed to the spin allowed ^1^MLCT *d* → *π* ^*^ transitions (assignment based on [32]). The molar absorption coefficients are presented in Table 1. The attachment of DCU moiety evidently decreases the intensity of the MLCT and LC bands while the other substituents at 4 and 4′ positions of the pyridine rings only slightly influence the energy and intensity of these bands.

**Table 1:** Photophysical properties for the ruthenium(II) complexes in air-equilibrated and deoxygenated aqueous solutions

	Absorption		Emission (air-equilibrated conditions)			Emission (deoxygenated conditions)	
	*λ*_max_ [nm]	ε [M^−1^ cm^−1^]	*λ*_max_ [nm]	*φ*	*τ* [ns]	*φ*	*τ* [µs]
[Ru(dip)_2_(bpy)]^2+^	278	126,300	613	0.0367 ± 0.0004	760 ± 10	0.1245 ± 0.0004	2.51 ± 0.01
	433	27,400					
	458	28,000					
[Ru(dip)_2_(CH_3_bpy-CH_3_)]^2+^	279	88,100	627	0.0254 ± 0.0009	690 ± 10	0.0778 ± 0.0016	2.23 ± 0.01
	438	19,000					
	461	18,500					
[Ru(dip)_2_(CH_3_bpy-COO)]^+^	279	92,200	622	0.0226 ± 0.0012	530 ± 10	0.0429 ± 0.0004	1.02 ± 0.01
	436	22,100					
	463	23,200					
[Ru(dip)_2_(CH_3_bpy-DCU)]^2+^	278	67,400	641	0.0044 ± 0.0002	460 ± 10	0.0049 ± 0.0002	1.85 ± 0.02
	440	15,700					
	463	16,200					
[Ru(dip)_2_(bpy-NitroIm)]^2+a^	278	88,400	621	0.0103 ± 0.0004	810 ± 10	0.0341 ± 0.0004	1.91 ± 0.01
	433	19,100					
	463	19,700					

^a^Data taken from [11]

The studied ruthenium complexes are found to be luminescent (Fig. S2) and they all express a shift of the emission maxima toward a longer wavelengths as well as a significant decrease in the quantum yield of luminescence with the respect to the parent complex [Ru(dip)_2_(bpy)]^2+^ (compare Table 1). The red shift is consistent with the withdrawing character of the attached substituents, which stabilize the lowest unoccupied molecular orbital of the bpy ligand, leading to a decrease in the energy of the MLCT level, responsible for the observed luminescence properties [14]. The reduction of luminescence quantum yield can be explained by “energy gap law”, predicting an increase of nonradiative transitions for lower and lower energy gaps between the emitting level and the ground state [14]. Also developing a side chain of bpy ligand as in case of bpy-2-nitroIm and bpy-DCU can cause an enhancement of the complexes mobility leading to the increase of the vibration mode of relaxation. This is also reflected by almost twofold reduction in a luminescence lifetime for [Ru(dip)_2_(CH_3_bpy-DCU)]^2+^ comparing to the parent complex. In contrast, for [Ru(dip)_2_(bpy-NitroIm)]^2+^ despite the decrease of the luminescence quantum yield, the luminescence lifetime increases probably due to interplay of the low-lying MLCT emitting levels and higher-lying nonemissive metal-centered levels [14]. The luminescence parameters of the tested compounds strongly depended on molecular oxygen concentration (2–3 times higher in the deoxygenated solution), since quenching of the emission can occur by the diffusion-controlled interaction and energy transfer between the triplet excited state of the metal complex and triplet ground state of oxygen. This opens a possibility for application of ruthenium complexes as a luminescence probe for the optical imaging of physiological hypoxia [10]. Only in the case of [Ru(dip)_2_(CH_3_bpy-DCU)]^2+^ oxygen does not influence strongly the quantum yield of luminescence, probably because of the increased complexes mobility and the vibration mode of relaxation caused by the expand ligand (bpy-DCU).

### Influence of macro-biomolecules on luminescence parameters of the ruthenium complexes

**Fig. 1:**
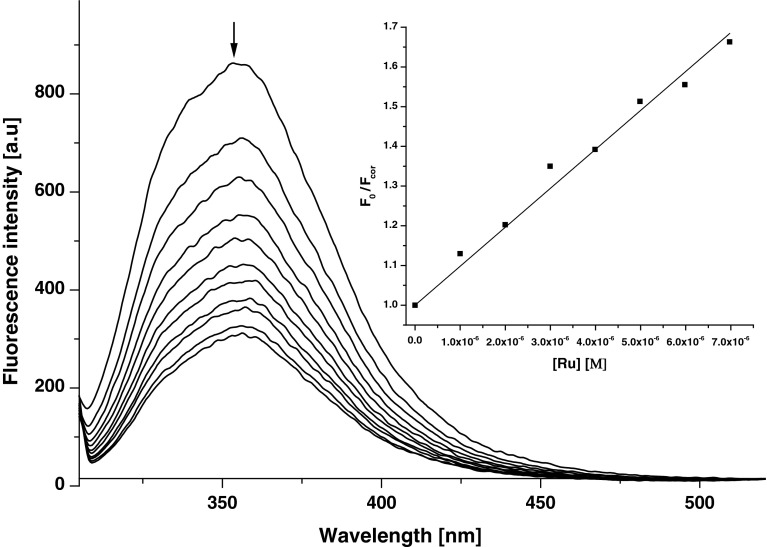
Fluorescence spectra of HSA in the present of different amount of [Ru(dip)_2_(CH_3_bpy-DCU)]^2+^. Insert: the Stern–Volmer plot showing the influence of the increasing concentration of ruthenium complex on the fluorescence intensity of HSA at *λ*_em_ = 358 nm. Experimental conditions: [HSA] = 1 μM; [Ru] = 0–10 μM; PBS pH 7.4; *λ*_ex_ = 295 nm, 37 °C

There has been a growing interest in the investigations of the interactions between metal complexes and biomolecules, since these interactions can alter compounds stability, distribution and cytotoxicity [33]. Recently, we have shown that the presence of human serum albumin (HSA) greatly influences the luminescence parameters of [Ru(dip)_2_(bpy-NitroIm)]^2+^ [11]. Similar effect but less pronounced (up to twofold enhancement) was observed for ruthenium complexes with one diimine and two phenathroline ligands with methyl groups substituted on position 3, 4, 7 and 8 [34]. HSA is the most abundant protein in blood and exerts significant impact on drugs transport and toxicity [29], it can also serve as a model for examination of the interaction between drugs and proteins. Albumin displays strong emission peak at 356 nm. Fluorescence is gradually decreased upon addition of ruthenium(II) polypyridyl complexes (as an example see Fig. 1).

The association constants for formation of adducts between ruthenium complexes and HSA were determined using spectrofluorimetric method [22, 35] (details are described in Supplementary Information) and are summarized in Table 2. Association constants for all complexes were found to be in the range of ca. 10^5^ M^−1^ suggesting a moderate interaction between the investigated ruthenium complexes and albumin. The parent complex [Ru(dip)_2_(bpy)]^2+^ is characterized by the weakest interaction with albumin, while the attachment of an additional moiety for bpy ligand increases affinity of the complexes towards HSA, probably by increasing the lipophilicity or the hydrophobic surface of the complexes. The exception is complex with bpy-DCU ligand that is probably too bulky to achieve efficient binding.

**Table 2:** The association constants for formation HSA–ruthenium(II) polypyridyl complex adducts

	*K*_a_ [×10^5^ M^−1^]
[Ru(dip)_2_(bpy)]^2+^	0.78 ± 0.02
[Ru(dip)_2_(CH_3_bpy-CH_3_)]^2+^	1.24 ± 0.06
[Ru(dip)_2_(CH_3_bpy-COO)]^+^	1.17 ± 0.04
[Ru(dip)_2_(CH_3_bpy-DCU)]^2+^	0.98 ± 0.03
[Ru(dip)_2_(bpy-NitroIm)]^2+^	1.10 ± 0.06

**Fig. 2:**
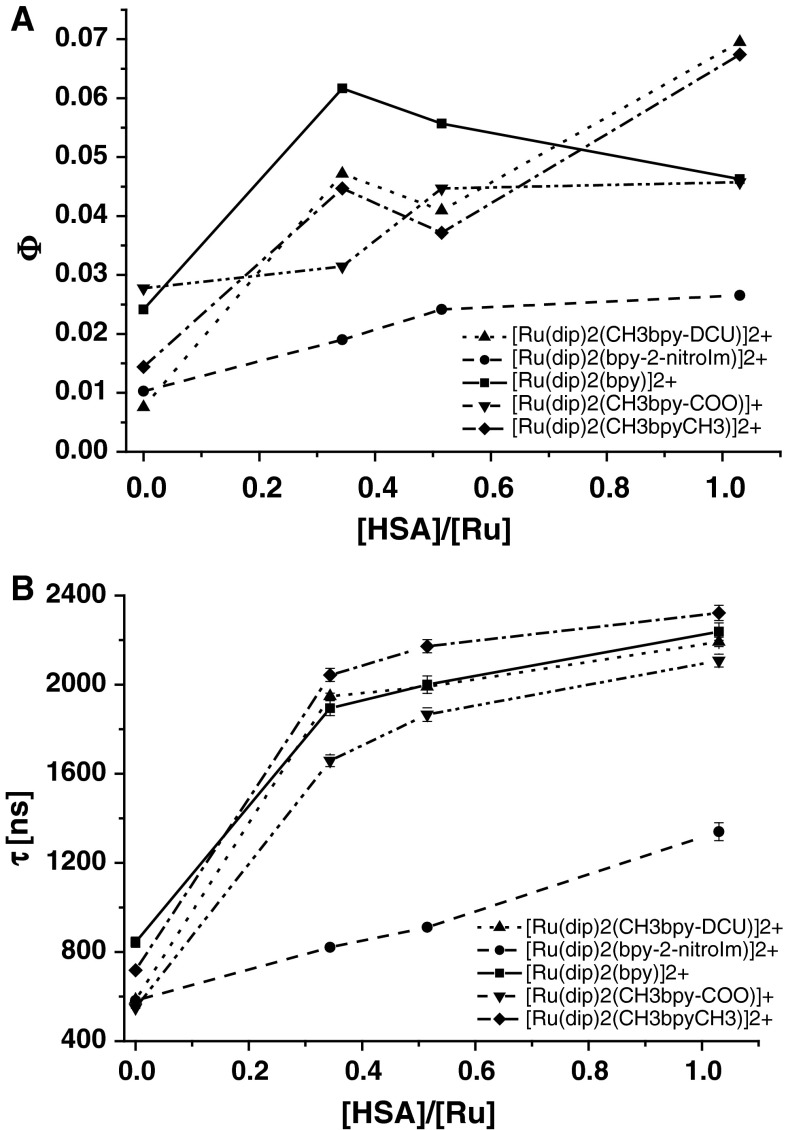
The luminescence quantum yield (**a**) and the average lifetime of luminescence (**b**) of the studied ruthenium complexes for various [HSA]/[Ru] ratios. Experimental conditions: [HSA] = 1 μM, [Ru] = 1–3 μM; PBS pH 7.4, excitation at 463 nm

The formation of adducts with albumin directly influences the luminescence parameters of the ruthenium complexes. The excitation of ruthenium complexes in the presence of increasing amount of albumin at 463 nm leads to gradual increase of both the luminescence quantum yield as well as the average lifetime of emission as shown in Fig. 2. It is very likely that the interaction of ruthenium complexes with HSA leads to partial separation of the substituents attached to bpy ligand from Ru center by protein scaffold. In this way protein can prevent from quenching its luminescence by these moieties. Moreover, the hydrophobic interactions may intensify the observed emission. One can assume that similar interaction occurs inside the cells with other proteins and causes the enhancement of the luminescence of the ruthenium complexes. [Ru(dip)_2_(CH_3_bpy-DCU)]^2+^ exhibits the largest increase in luminescence parameters upon addition of HSA: in PBS in the absence of addition *φ* = 0.0076 while at [HSA]/[Ru] = 1, *φ* = 0.0472. This gives ca. ninefold increase in quantum yield of luminescence and makes HSA–[Ru(dip)_2_(CH_3_bpy-DCU)]^2+^ adduct the most luminescent species among the studied protein–ruthenium complex adducts. Correspondingly, the luminescence average lifetime of ruthenium complexes upon addition of HSA changes in a similar way, showing 2- to 4-fold increase at [HSA]/[Ru] = 1 (Fig. 2b).

The investigated ruthenium complexes have moderate DNA-binding constants (see Table 3, for experimental details see Supporting Information and Fig. S3). The intercalation of these complexes is not expected since unlike dppz ligand the dip ligands are too bulky to permit efficient intercalation or close contact while bpy ligands are too small to enable significant stacking [15] and the selected substituents do not influence strongly the binding properties. The type of interaction between [Ru(dip)_2_(bpy-2-nitroIm)]^2+^ and DNA was thoroughly studied elsewhere [11]. Based on our previous research, we can suggest that electrostatic interaction and binding through a DNA groove are responsible for DNA–Ru interaction for tested complexes. The interaction with DNA causes unusual change in luminescence properties of these complexes (Fig. S4). Unlike ruthenium “light switch” complexes ([RuL_2_(dppz)]^2+^), after addition of small excess of DNA luminescence of [Ru(dip)_2_(R_1_bpy-R_2_)]^2+/+^ complexes decreases as a result of diffusion quenching by DNA [11]. Interestingly, [Ru(dip)_2_(CH_3_bpy-DCU)]^2+^ does not exhibit quenching of luminescence, but only small shift towards lower wavelengths (Fig. S4). It is reasonable to assume, that its weak luminescence properties arising from an increased mobility of the complex caused by the attached expand ligand (bpy-DCU) making it no longer sensitive toward quenchers like DNA or O_2_ (Table 1). Higher DNA/Ru ratio(>80 excess of DNA) significantly increases of ruthenium complexes luminescence intensity (Fig. S4). This can be explained by reduction of their mobility and the vibration mode of relaxation, as well as the protection of the ruthenium complexes from quenching by water molecules due to the hydrophobic environment inside the DNA [36].

**Table 3:** The binding constants for formation of DNA–ruthenium(II) polypyridyl complex adducts

	*K*_b_ [×10^5^ M^−1^]
[Ru(dip)_2_(bpy)]^2+^	0.82 ± 0.01
[Ru(dip)_2_(CH_3_bpy-CH_3_)]^2+^	0.53 ± 0.03
[Ru(dip)_2_(CH_3_bpy-COO)]^+^	1.05 ± 0.15
[Ru(dip)_2_(CH_3_bpy-DCU)]^2+^	0.60 ± 0.02
[Ru(dip)_2_(bpy-NitroIm)]^2+^	0.68 ± 0.01

### Lipophilicity of the ruthenium polypyridyl complexes

Lipophilicity is commonly described as the *n*-octan-1-ol/water partition coefficient (expressed in log *P*_o/w_) of the compounds, which was determined by a shaking method. It is well known that lipophilicity of the metal complexes is critical for their cellular selective uptake: cationic probes showing uptake in nuclei and lysosomes have −5 < log *P*_o/w_ < 0, while dyes with 0 < log *P*_o/w_ < 5 accumulate preferentially in mitochondria and endoplasmic reticulum [6]. The log *P*_o/w_ values of ruthenium(II) polypyridyl complexes are listed in Table 4. The additional substituents in bpy ligand increase lipophilicity of the ruthenium complexes. Among dicationic complexes [Ru(dip)_2_(CH_3_bpy-DCU)]^2+^ is characterized by the highest log *P*_o/w_ value arising from the expanded substituent in bpy ligand, while for [Ru(dip)_2_(CH_3_bpy-COO)]^+^the relatively high log *P*_o/w_ is caused by monocationic character of the complex. The literature data have demonstrated that positively charged ruthenium complexes show higher uptake than neutral one [15], at the same time monocationic species show higher uptake compared to the dicationic one [37]. However, the lipophilicity of the compound outweighs the influence of the number of positive charges [37]. The cellular uptake is at least partially controlled by lipophilicity, therefore this parameter can also influence the cytotoxicity of ruthenium complexes as well as the intensity of the observed luminescence signal.

**Table 4:** Lipophilicity (log *P*_o/w_ values) of the ruthenium(II) complexes

	log *P*_o/w_
[Ru(dip)_2_(bpy)]^2+^	0.328 ± 0.026
[Ru(dip)_2_(CH_3_bpy-CH_3_)]^2+^	0.484 ± 0.046
[Ru(dip)_2_(CH_3_bpy-COO)]^+^	1.857 ± 0.079
[Ru(dip)_2_(CH_3_bpy-DCU)]^2+^	1.114 ± 0.012
[Ru(dip)_2_(bpy-NitroIm)]^2+^	0.413 ± 0.083

### In vitro cytotoxicity

The cytotoxicity of the ruthenium complexes was evaluated using 4T1 breast cancer cell line. Cisplatin was used as a positive control. Ruthenium complexes can interact with proteins [34, 38], so cytotoxicity of the complexes was evaluated both in medium with or without serum. The IC_50_ values of the tested complexes are listed in Table 5.

**Table 5:** The IC_50_ values of the ruthenium(II) complexes and cisplatin against 4T1 cell line after 24 h of incubation in medium with or without serum (2 %)

	Without serum	With serum
[Ru(dip)_2_(bpy)]^2+^	6.79 ± 1.09	13.56 ± 1.75
[Ru(dip)_2_(CH_3_bpy-CH_3_)]^2+^	4.90 ± 0.30	9.32 ± 1.37
[Ru(dip)_2_(CH_3_bpy-COO)]^+^	8.66 ± 1.47	13.90 ± 3.00
[Ru(dip)_2_(CH_3_bpy-DCU)]^2+^	4.71 ± 0.18	9.01 ± 1.33
[Ru(dip)_2_(bpy-NitroIm)]^2+^	10.64 ± 1.05	18.78 ± 1.29
Cisplatin	73.00 ± 14.94	59.81 ± 8.32

Ruthenium polypyridyl complexes are found to be much more cytotoxic than cisplatin against 4T1 cell line, ca. one order of magnitude. Among the tested compounds [Ru(dip)_2_(CH_3_bpy-DCU)]^2+^ and [Ru(dip)_2_(CH_3_bpy-CH_3_)]^2+^ were found to be the most toxic in the studied conditions. One of the possible explanations is the high lipophilicity of the former one and moderate liphophilicity and smaller size of the later one, which should facilitate their uptake. [Ru(dip)_2_(CH_3_bpy-COO)]^+^ is less cytotoxic despite the highest lipophilicity, this may be due to its lower accumulation as confirmed by uptake studies. The addition of serum containing as a major component bovine serum albumin to the incubation medium results in ca. twofold decrease in cytotoxicity (see Table 5). Likely the formed adducts between the tested ruthenium compounds and serum proteins are less accessible for the cells. Consequently, ruthenium accumulation may get less efficient. The range of cytotoxicity reduction correlates with the values of the protein–Ru association constants (Table 2). The same cytotoxic order for the studied ruthenium complexes was also found in human lung adenocarcinoma (A549) cell line as well as in two endothelial cell lines (murine lung microvascular endothelial and murine endothelial cells from AGM region from 10.5 dpc embryos, details are presented in Supplementary information and in Table S1).

**Fig. 3:**
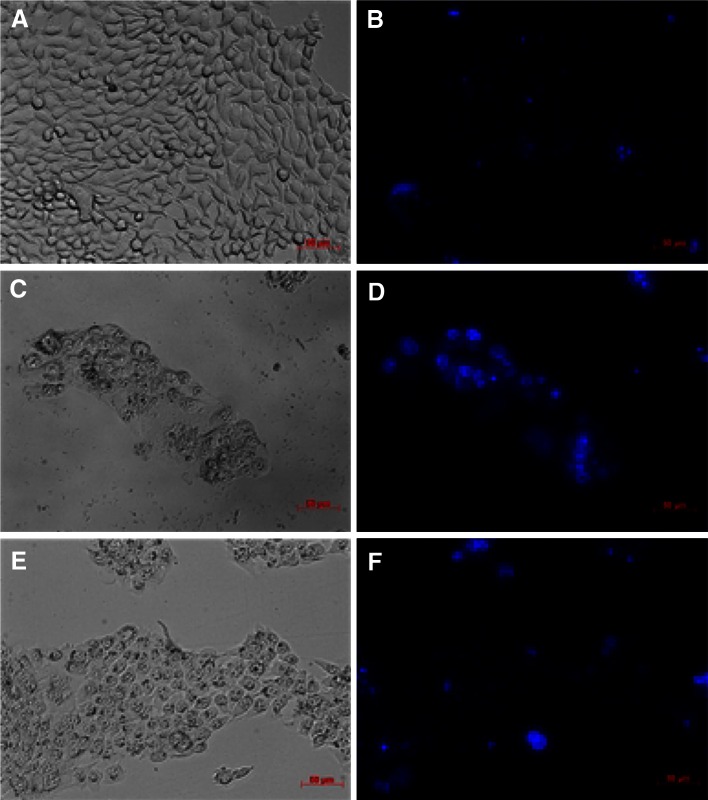
Effect of the ruthenium complexes treatment towards mammary cancer cells. **a**, **c**, **e**: DIC (differential interferential contrast), **b**, **d**, **e**: fluorescence labeling of the nuclei by Hoechst 33258 of the 4T1 cells after treatment by 8 µM [Ru(dip)_2_(CH_3_bpy-DCU)]^2+^ (**c**, **d**) or [Ru(dip)_2_(bpy)]^2+^ (**e**, **f**), for 24 h. **a** and **b** show control cells

In order to clarify how ruthenium complexes affected cell growth, after treatment with compound cells were examined by fluorescence microscope. Representative images are shown in Fig. 3. 4T1 cells after incubation with ruthenium compounds show marked morphological sign of apoptosis, such as decreasing amount of detached cells, cells rounding and shrinkage [39]. To evaluate the nucleus morphological changes, cells were stained with Hoechst and analyzed by fluorescence microscopy. The untreated population of cells displays a homogenous morphology with round nuclei evenly stained with Hoechst. After treatment with ruthenium complexes, most of the cells display fragmented nuclei with densely stained nucleus granular bodies of chromatin (so-called “apoptotic bodies”) [29]. The results suggest that cytotoxic effect of ruthenium complexes is based at least partially on their pro-apoptotic properties.

### Cellular uptake studies by ICP-MS

**Fig. 4:**
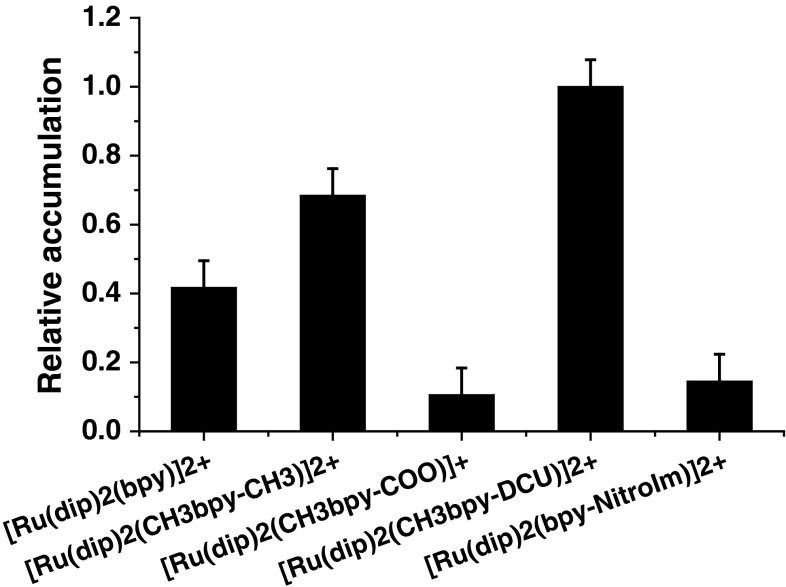
Relative ruthenium accumulation in 4T1 cell line in a single cell determined by ICP-MS. Experimental conditions: [Ru-complex] = 2 µM, 24 h incubation in the darkness

To quantify the concentration of ruthenium accumulated inside the 4T1 cells, the ICP-MS measurements were used. The uptake was determined for the sub-lethal dose of ruthenium compounds to evaluate the ability of the tested complexes to internalize into live cells. The absolute values of ruthenium concentration found in cells strongly depend on applied experimental conditions [40], thus the obtained results shown in Fig. 4 are presented in relation to [Ru(dip)_2_(CH_3_bpy-DCU)]^2+^ that exhibits the highest accumulation. The actual values of the accumulated ruthenium concentration vary from 130 to 1,200 µM (Fig. S5) and it correlates with previously reported ICP-MS measurement of ruthenium complexes uptake [31]. The rise of the accumulation for dicationic ruthenium complexes correlates with their increased lipophilicity. Such relationship has been already reported [41]. The monocationic [Ru(dip)_2_(CH_3_bpy-COO)]^+^ expresses much smaller accumulation than the rest of the studied complexes despite having the highest log *P*_o/w_ value. The charge of the compound displays a stronger influence than lipophilicity on ruthenium accumulation. This can arise from a possible facilitated transport of ruthenium polypyridyl complexes into cells via passive diffusion due to membrane potential [31]. The greater uptake the higher cytotoxicity suggests that internalization of the ruthenium complexes is required for their biological activity.

### Cell luminescence upon treatment by ruthenium complexes

**Fig. 5:**
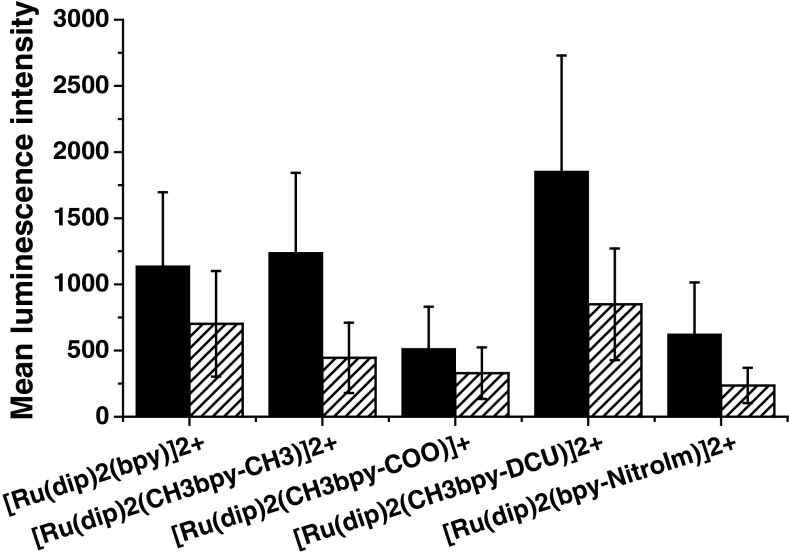
Mean luminescence intensity of 4T1 cell lines incubated with ruthenium compounds measured by flow cytometry (*filed* incubated without serum, *dashed* incubated with serum). Experimental conditions: [Ru-complex] = 2 µM, 24 h of incubation in medium without or with serum (2 %)

The uptake of the tested ruthenium complexes by 4T1 cells was also monitored using flow cytometry (Fig. S6). It must be noted that the light emission by the cells was not proportional to the amount of ruthenium incorporated inside cells since the luminescence quantum yield for various ruthenium complexes is different. Furthermore, the luminescence of ruthenium complexes is substantially influenced by the interaction with proteins. The observed luminescence signal (shown in Fig. 5) combines both the ability of the compounds to cross the cell membrane and the luminescence intensity emitted after interaction with cytoplasmic molecules and organelles. This method is adequate for an evaluation of the cell staining capacity by ruthenium complexes. [Ru(dip)_2_(CH_3_bpy-DCU)]^2+^ provided the highest luminescence of 4T1 cells. This complex is characterized by the smallest quantum yield of luminescence, but due to its interaction with protein the quantum yield greatly increases and its high lipophilicity intensifies its uptake. In general, for all the studied complexes the order of luminescence expressed by cells correlates with the cellular uptake determined by ICP-MS method as well as the cytotoxicity data. When the incubation of ruthenium complexes with cells was carried out in medium supplemented with serum, the cells expressed smaller luminescence intensity (see Fig. 5). This further confirms that the access of the ruthenium complexes to cells is lower, probably due to formation of adduct with proteins. Similar results have been obtained for endothelial MLuMEC cells (Supplementary Information Fig. S7).

**Fig. 6:**
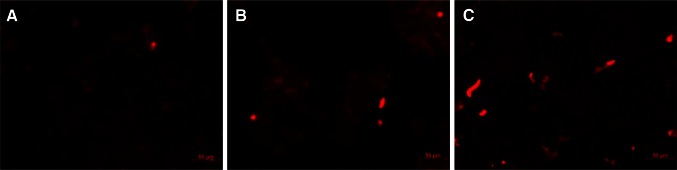
Fluorescence images of cells incubated with 1 µM [Ru(dip)_2_(CH_3_bpy-DCU)]^2+^ (**a**), [Ru(dip)_2_(bpy)]^2+^ (**b**), [Ru(dip)_2_(bpy-NitroIm)]^2+^ (**c**) for 24 h

Analyzing the staining pattern of live cells for the studied ruthenium complexes, no significant differences between each other is found (Fig. 6; Fig. S8), only the intensity of the staining is altered. This suggests that the place of accumulation of ruthenium complexes is determined by two dip ligands attached to each studied complex. Cytoplasm is marked homogeneously with edge of the nucleus (mitochondria/endoplasmic reticulum) pointed out. No staining in the nucleus is observed confirming no incorporation in the DNA.

## Conclusions

We have shown for a series of complexes [Ru(dip)_2_(R_1_bpy-R_2_)]^2+/+^ that the higher luminescence parameters measured in aqueous solution do not directly correspond to the best cell staining properties. It is a combined effect of the luminescence expressed in cellular environment and the extent of its accumulation. For all studied complexes the interaction with human serum albumin results in pronounced increase of quantum yield and lifetime of luminescence. This phenomenon can be called “light switch” effect and for the studied complexes is observed only in the presence of albumin while DNA induces minor changes in their luminescence. As the intracellular protein content is high (ca. 50–400 mg/ml) we can expect that similar effect is taking place. Therefore, while designing cellular probes it is necessary to take into account not only the luminescence parameters of a single substance, but also the possible resulting adducts with biomacro molecules. The [Ru(dip)_2_(CH_3_bpy-DCU)]^2+^ regardless of its weakest luminescence parameters displays the best staining properties. Additionally, the lipophilicity and complex charge determine the level of its uptake which explains its cytotoxicity and imaging properties. The mechanism of action of these type of complexes remains still unknown, but our preliminary data point out that they can induce the apoptosis.

## Electronic supplementary material

Below is the link to the electronic supplementary material.

Supplementary material 1 (PDF 953 kb)
