# Supplementary material for: Multifaceted interplay between lipophilicity, protein interaction and luminescence parameters of non-intercalative ruthenium(II) polypyridyl complexes controlling cellular imaging and cytotoxic properties
Source: J Biol Inorg Chem. 2014 Aug 26;19(8):1305–16. doi: 10.1007/s00775-014-1187-5 (PMC4240912; doi:10.1007/s00775-014-1187-5)
Supplement: Supplementary file 1 — Supplementary material 1 (PDF 953 kb) [file 775_2014_1187_MOESM1_ESM.pdf]

To be submitted to *Journal of Biological Inorganic Chemistry*

Supplementary information

Multifaceted interplay between lipophilicity, protein interaction and luminescence parameters of non-intercalative ruthenium(II) polypyridyl complexes controlling cellular imaging and cytotoxic properties.

Olga Mazuryk[a], Katarzyna Magiera[a,b], Barbara Rys[b], Franck Suzenet[c], Claudine Kieda[d], Małgorzata Brindell[a,✉]

[a] *Department of Inorganic Chemistry, Faculty of Chemistry, Jagiellonian University, Ingardena 3, 30-060 Krakow, Poland*

[b] *Department of Organic Chemistry, Faculty of Chemistry, Jagiellonian University, Ingardena 3, 30-060 Krakow, Poland*

[c] *Institute of Organic and Analytical Chemistry, University of Orléans, UMR-CNRS 7311, rue de Chartres, BP 6759, 45067 Orléans Cedex 2, France*

[d] *Centre de biophysique moléculaire, CNRS, rue Charles Sadron, 45071, Orléans Cedex, France*

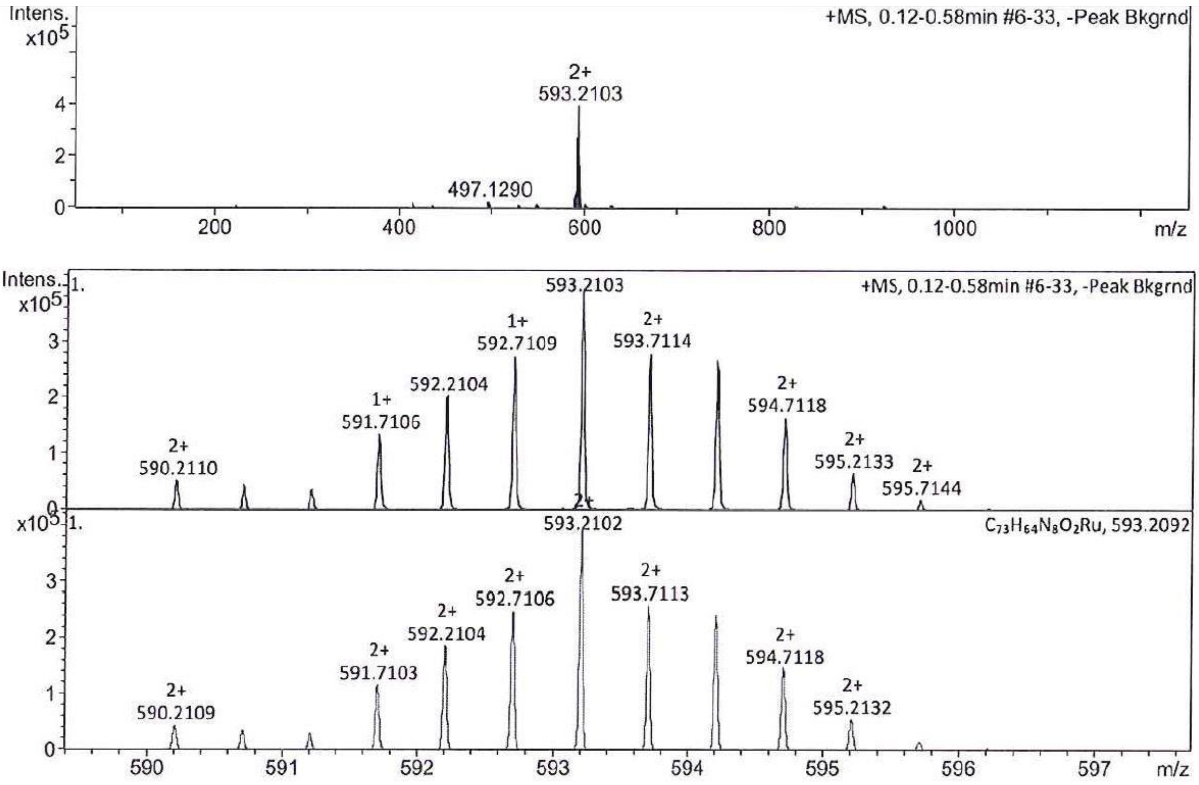

Fig. S1A. HRMS spectra for $[Ru(dip)_2(CH_3bpy\text{-}DCU)]^{2+}$

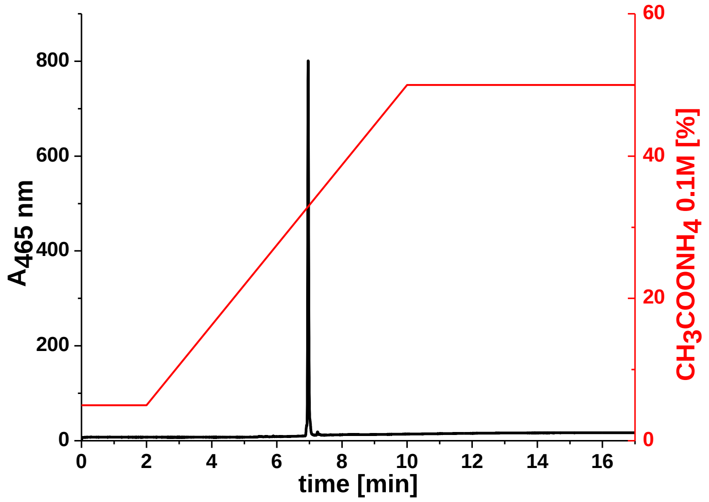

Fig. S1B. The chromatogram for the $[Ru(dip)_2(CH_3bpy\text{-}DCU)]^{2+}$

Equipment: Perkin Elmer Flexar with diode-array detector, column oven
Column: Alltima™ HP HILIC (5 µm, 4.6 x 150 mm)
Solvent system: 80 % of A (2 min) to 60 % (5 min)
Mobile A: acetonitrile
Mobile B: 0.1 M ammonium acetate
Flow rate: 2 ml/min
Temperature: 30 °C
Detection wavelength: 465 nm

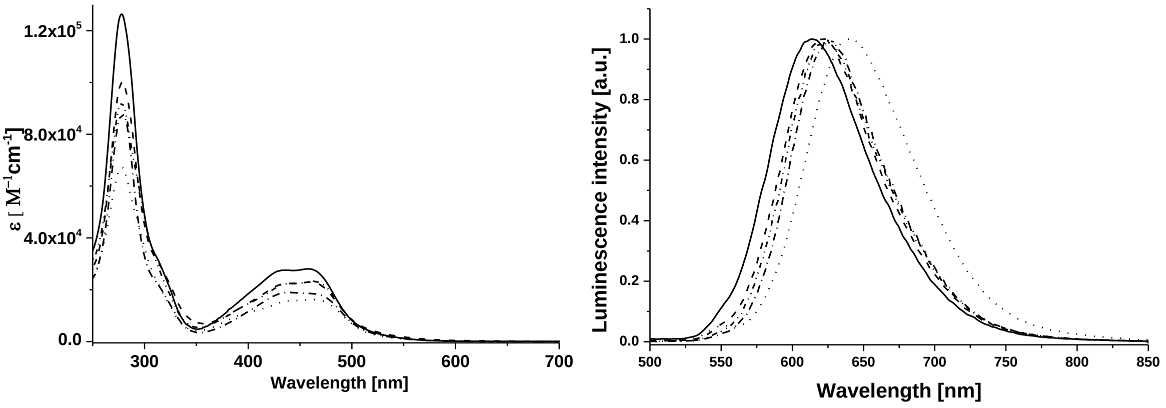

Fig. S2. Absorption (A) and emission (B) spectra (excitation at 463 nm) of $[Ru(dip)_2(bpy)]^{2+}$ (—), $[Ru(dip)_2(bpy\text{-}nitroIm)]^{2+}$ (– – –), $[Ru(dip)_2(CH_3bpy\text{-}DCU)]^{2+}$ (· · ·), $[Ru(dip)_2(CH_3bpy\text{-}COO)]^{+}$ (– · ·) and $[Ru(dip)_2(CH_3bpy\text{-}CH_3)]^{2+}$ (– ·) in H_2O.

Determination of association constants for formation of HSA-Ru-complex adducts

Human serum albumin (HSA) consists of 585 amino acids residues, organized in 3 domains.[1] HSA possesses single tryptophan residue Trp214 located in subdomain IIA. This residue is responsible for the majority of the intrinsic fluorescence of human albumin.[2]

When molecules are bound to HSA, the change in fluorescence of HSA (Trp214) is induced ether by quenching by binding of molecules in close proximity of the residue or by conformational change of protein caused by the binding or other interactions. Albumin displays strong emission peak at 356 nm, which is diminished upon addition of ruthenium(II) polypyridyl complexes. The contribution to the observed emission from ruthenium complex is marginal. The Stern-Volmer equation has been employed for quantitative analysis of the observed quenching:

$$\frac{F_0}{F} = 1 + k_q \tau_0 \cdot [Q] = 1 + K_{SV} \cdot [Q]$$

where F_0 and F are the fluorescence intensities of HSA in the absence and the presence of quencher (Q, Ru complex), respectively; k_q is the bimolecular quenching constant; τ_0 is the average lifetime of the HSA in the absence of quencher (7×10^{-9} s [3]), $[Q]$ is the concentration of quencher, K_{SV} is the Stern–Volmer constant. Since ruthenium complexes both absorb light at the excitation and the emission wavelengths in order to correlate the inner filter effect the following equation was used:

$$F_{cor} = F \cdot 10^{\frac{A_{em} + A_{ex}}{2}}$$

where F_{cor} and F are the corrected/uncorrected fluorescence intensities of HSA, A_{em} and A_{ex} are the absorbance value of the solution at the emission and the excitation wavelength respectively.

The calculated value of K_{SV} was found to be ≈ 10^5 M^{-1} at 37 °C. The bimolecular quenching constant at 37 °C was calculated ≈ 10^{13} M^{-1} s^{-1} suggesting static mechanism of quenching [2] involving some type of binding interaction between HSA and ruthenium complexes. In this case the Stern-Volmer constant can be consider as an association constant for formation of HSA-Ru-complexes adducts.

Interaction of ruthenium complexes with calf thymus DNA (ct-DNA)

The absorption titration experiment is one of the most useful and popular ways to study the DNA-binding properties of metal complexes. The addition of the ct-DNA to the constant ruthenium complexes concentration up to [DNA]/[Ru] ratio of 2 results in a gradual decreasing of absorption for both LC and MLCT bands (examples are shown in Fig. S3). The observed hypochromism and rather small bathochromism at MLCT absorption band, suggests that ruthenium complex interacts with DNA in a specific way, possibly through

intercalation.[4] The hypochromism and batochromism at MLCT bands is found usually higher for other reported polypyridyl DNA intercalative Ru(II) complexes.[5-8] To assess this interaction the intrinsic DNA-binding constant was calculated from the following equation [4]:

$$\frac{[DNA]}{\varepsilon_a - \varepsilon_f} = \frac{[DNA]}{\varepsilon_b - \varepsilon_f} + \frac{1}{K_b(\varepsilon_b - \varepsilon_f)}$$

where [DNA] is the total DNA concentration in nucleotides, ε_a, ε_b, ε_f are the apparent absorption coefficients of A/[ruthenium complex] of the MLCT absorption band at a given DNA concentration, fully bound and free ruthenium complex, respectively, K_b is binding constant. The calculated binding constants are presented in **Table 3**.

Interestingly, the increasing of the [DNA]/[Ru] ratio more than 2 leads to loss of the isosbestic points and gradual decrease of the absorption of MLCT bands. This observation suggests that some other type of non-specific interaction can also occur. However due to rather week interaction with DNA it is not further discussed.

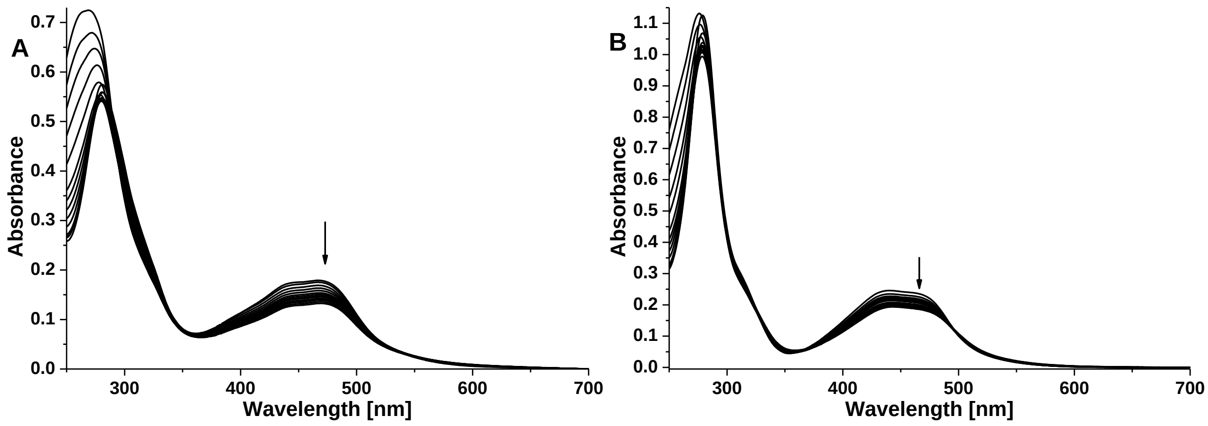

Fig. S3. Changes in absorption spectra of $[Ru(dip)_2(CH_3bpy\text{-}DCU)]^{2+}$ and $[Ru(dip)_2(CH_3bpy\text{-}CH_3)]^{2+}$ after addition of ct-DNA. Experimental conditions: [Ru] = 10 μM; Tris/HCl pH 7.4, T = 37°C, A. [DNA] = 0 – 2 μM; B. [DNA] = 5 – 120 μM.

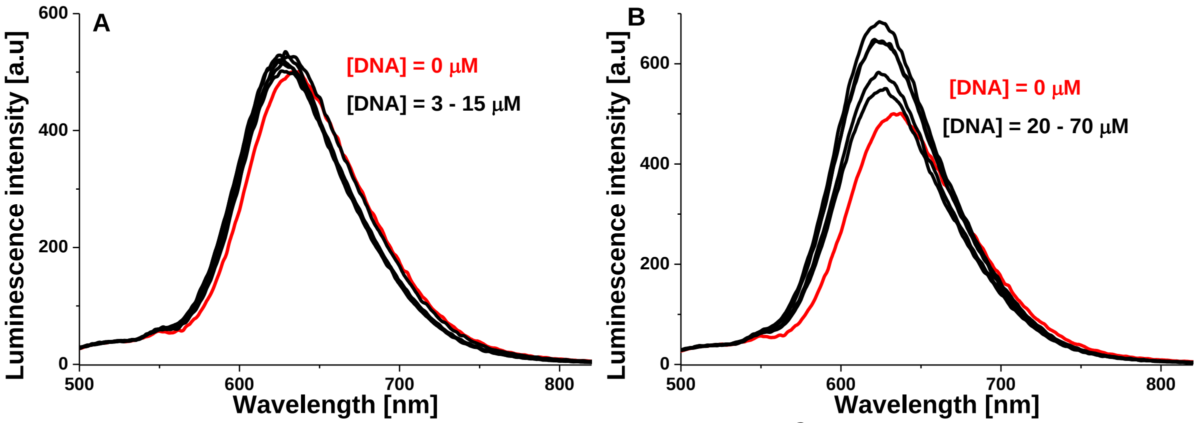

Fig. S4. Luminescence spectra of $[Ru(dip)_2(CH_3bpy\text{-}DCU)]^{2+}$ after addition of ct-DNA. A. [DNA] = 0 – 15 μM and B. [DNA] = 20 – 70 μM. Experimental conditions: [Ru] = 3 μM; Tris/HCl pH 7.4, T = 37 °C.

Cytotoxicity study

FVB mouse lung microvascular endothelial cells (MLuMEC FVB) and murine endothelial cells isolated from aorta-gonad-mesonephros (AGM) region of 10.5 dpc embryos (MAgEC 10.5) were cultured in OptiMEM with Glutamax-I (Gibco Invitrogen) supplemented with 2% fetal bovine serum (FBS), 0.4% gentamycin and 0.2% fungizone. Human lung adenocarcinoma epithelial cell line (A549) was cultured in DMEM (Gibco Invitrogen) with 10% FBS, 1% peniciline and streptomycin and 0.2% fungizone. Cells were routinely cultured at 37 °C in a humidified incubator in 5% CO_2 atmosphere.

Cell viability was measured using Alamar Blue assay. Cells were seeded on 96 wells plate with density of 1×10^4 cells per cm^2 and cultured for 1 day. Then cells were incubated with various concentrations of compounds in medium with or without 2% FBS for 24 and 48 h under normoxic and hypoxic conditions in the darkness. Next cells were washed with PBS and incubated in Alamar Blue solution for 3 h. The cell viability was quantified at 605 nm using 560 nm excitation light (VICTOR 3V multilabel plate reader PerkinElmer or Tecan Infinite200 Reader). Experiments were performed in triplicates and each experiment was performed at least three times to get the mean values ± standard derivation. The viability was calculated with regard to the untreated cells control. The IC_{50} values were determined using Hill equation (Origin 9.0) [9].

$$y = y_0 + \frac{(y_{100} - y_0)[c]^H}{[IC_{50}]^H + [c]^H}$$

Table S1. The IC_{50} values of the ruthenium(II) complexes and cisplatin against cancer (A549) and endothelial (MLuMEC, MAgEC 10.5) cell lines after 24 h of incubation in medium with or without serum (2%).

[μM]	**A549**		**MLuMEC**		**MAgEC 10.5**	
	**without serum**	**with serum**	**without serum**	**with serum**	**without serum**	**with serum**
$[Ru(dip)_2(bpy)]^{2+}$	13.50±5.3	27.70±1.2	4.70±0.41	11.73±1.6	4.15±0.57	16.67±1.88
$[Ru(dip)_2(CH_3bpy-CH_3)]^{2+}$	5.47±0.87	11.58±2.19	3.36 ± 0.35	8.32 ± 0.06	2.45 ± 0.42	6.28 ± 0.52
$[Ru(dip)_2(CH_3bpy-COO)]^{+}$	6.05±1.87	9.79±0.89	7.74 ± 1.26	18.07±2.19	7.23 ± 0.53	15.96 ± 1.50
$[Ru(dip)_2(bpy-NitroIm)]^{2+}$	11.80±1.20	17.50±5.70	6.08±0.70	10.39±1.31	4.65±0.62	7.40±0.81
$[Ru(dip)_2(CH_3bpy-DCU)]^{2+}$	5.43±0.11	7.22±0.34	2.28 ± 0.61	5.84 ± 0.24	2.54 ± 1.12	4.62 ± 0.22
cisplatin	125.99±30.50	71.31±22.93	24.63±2.89	14.54±1.40	9.38±0.67	8.75±0.20

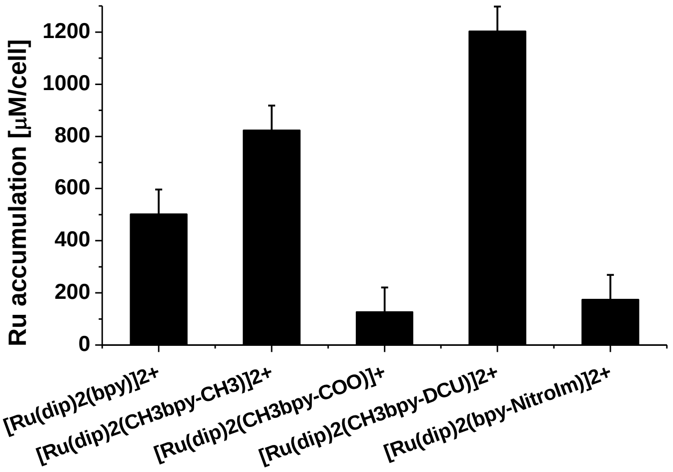

Fig. S5. Ruthenium accumulation in 4T1 cell line in a single cell determined by ICP-MS. Experimental conditions: [Ru-complex] = 2 μM, 24 h incubation in the darkness.

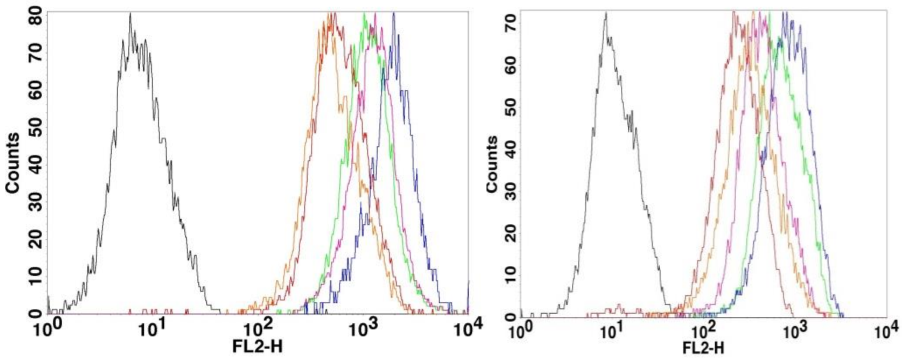

Fig. S6. Flow cytometry histograms of 4T1 cells incubated with ruthenium complexes 2 μM ($[Ru(dip)_2(bpy)]^{2+}$ (green), $[Ru(dip)_2(bpy\text{-}nitroIm)]^{2+}$ (red), $[Ru(dip)_2(CH_3bpy\text{-}DCU)]^{2+}$ (blue), $[Ru(dip)_2(CH_3bpy\text{-}COO)]^{+}$ (orange) and $[Ru(dip)_2(CH_3bpy\text{-}CH_3)]^{2+}$ (pink) incubated in medium with (B) or without serum (A) for 24 hours (black line – control cells).

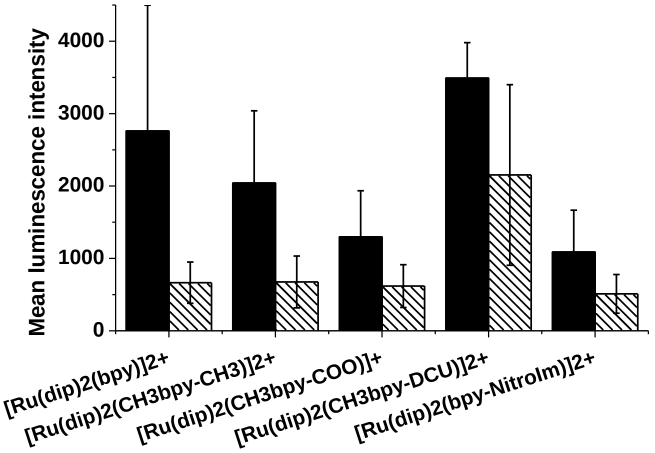

Fig. S7. Mean luminescence intensity of MLuMEC cell lines incubated with ruthenium compounds measured by flow cytometry (filed – incubated without serum, dashed – incubated with serum). Experimental conditions: [Ru-complex] = 2 μM, 24 h of incubation in medium without or with serum (2%).

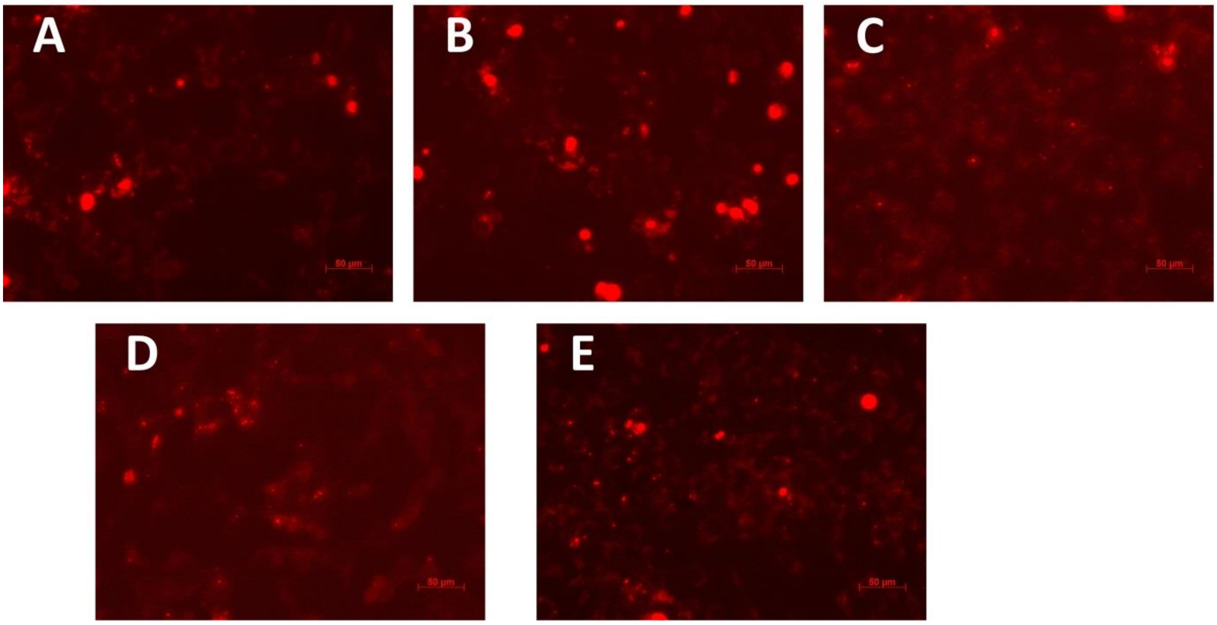

Fig. S8A. Fluorescence images of MLuMEC cells incubated with 2 μM [Ru(dip)$_2$(bpy)]$^{2+}$ (A), [Ru(dip)$_2$(CH$_3$bpy-CH$_3$)]$^{2+}$ (B), [Ru(dip)$_2$(CH$_3$bpy-COO)]$^{+}$ (C), [Ru(dip)$_2$(bpy-NitroIm)]$^{+}$ (D) and [Ru(dip)$_2$(CH$_3$bpy-DCU)]$^{2+}$ (E) for 24 h.

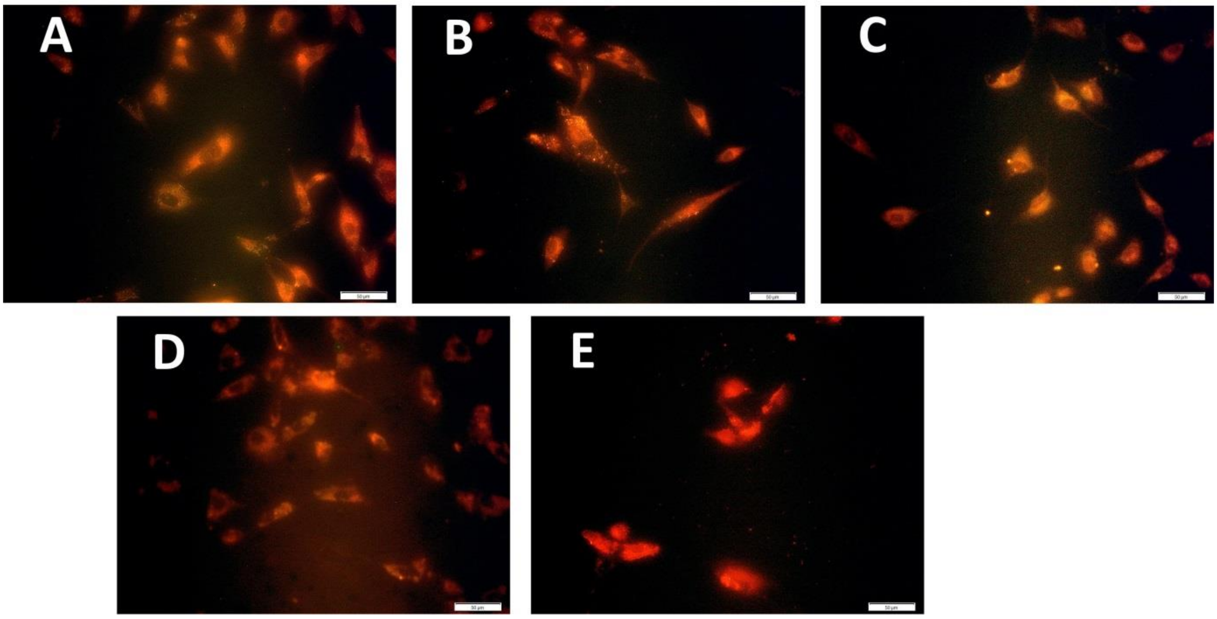

Fig. S8B. Fluorescence images of A549 cells incubated with 2 μM [Ru(dip)$_2$(bpy)]$^{2+}$ (A), [Ru(dip)$_2$(CH$_3$bpy-CH$_3$)]$^{2+}$ (B), [Ru(dip)$_2$(CH$_3$bpy-COO)]$^{+}$ (C), [Ru(dip)$_2$(bpy-NitroIm)]$^{+}$ (D) and [Ru(dip)$_2$(CH$_3$bpy-DCU)]$^{2+}$ (E) for 24 h.

References

[1] X.M. He, D.C. Carter, (1992), Nature, 358: 209-215.

[2] J.R. Lakowicz (2006), Principles of Fluorescence Spectroscopy, Springer, New York.
[3] O. Mazuryk, E. Niemiec, G. Stochel, I. Gillaizeau, M. Brindell, (2013), J. Lumin., 140: 51-56.
[4] A. Srishailam, Y.R. Kumar, N.M.D. Gabra, P.V. Reddy, N. Deepika, N. Veerababu, S. Satyanarayanna, (2013), J. Fluoresc., 23: 897-908.
[5] B. Sun, Y.C. Wang, C. Qian, J. Chu, S.M. Liang, H. Chao, L.N. Ji, (2010), J. Mol. Struct., 963: 153-159.
[6] X.W. Liu, S.B. Zhang, L. Li, Y.D. Chen, J.L. Lu, (2013), J. Organomet. Chem, 729: 1-8.
[7] L. Blackmore, R. Moriarty, C. Dolan, K. Adamson, R.J. Forster, M. Devocelle, T.E. Keyes, (2013), Chem. Commun., 49: 2658-2660.
[8] C. Wang, Q. Yu, L. Yang, Y. Liu, D. Sun, Y. Huang, Y. Zhou, Q. Zhang, J. Liu, (2013), Biometals, 26: 387-402.
[9] J. Weyermann, D. Lochmann, Z. A., (2005), Int. J. Pharm., 288: 369-376.
